# Predictors of Asylum Seekers’ Health Care Utilization in the Early Phase of Resettlement

**DOI:** 10.3389/fpsyt.2020.00475

**Published:** 2020-05-28

**Authors:** David Kindermann, Valentina Zeyher, Ede Nagy, Hans-Christoph Friederich, Kayvan Bozorgmehr, Christoph Nikendei

**Affiliations:** ^1^Department of General Internal Medicine and Psychosomatics, University of Heidelberg, Heidelberg, Germany; ^2^Department of General Practice and Health Services Research, University Hospital Heidelberg, Heidelberg, Germany; ^3^Department of Population Medicine and Health Services Research, School of Public Health, Bielefeld University, Bielefeld, Germany

**Keywords:** asylum seekers, health care utilization, sense of coherence, suppression, reappraisal

## Abstract

**Background:**

Asylum seekers display high prevalence rates of posttraumatic stress disorder, depression, anxiety, and panic disorder due to pre-, peri-, and post-migration stressors. In contrast to the high mental health burden, health care utilization among asylum seekers in the early phase of resettlement is low. However, the early stages after migration are a particularly vulnerable phase in which psychosocial support measures are needed to prevent mental disorders from becoming chronic.

**Objective:**

To identify predictors of asylum seekers’ health care utilization in the early stages of resettlement.

**Methods:**

Using hierarchical logistic regression analysis, the variance explanation of the (1) general utilization of health care services as well as the individual utilization of (2) outpatient psychiatrists, (3) counselling centers, and (4) general practitioners was analyzed in *n* = 65 asylum seekers. A structured interview on health care utilization took place between three to five months after assessment of possible predictors. We defined the following three groups of predictors a) the sociodemographic variables gender, age, number of children, religion, language proficiency, b) the psychological variables sense of coherence and emotion regulation as well as c) the asylum seekers’ psychiatric diagnoses.

**Results:**

Individual sociodemographic factors, such as gender, age, and number of children as well as the emotion regulation strategy of expressive suppression and sense of coherence were shown to be predictive for the utilization of health care services among asylum seekers.

**Conclusions:**

Low-threshold, culture-sensitive treatment offers for asylum seekers should be established in the early phase after migration. General practitioners should be a central hub for further referrals to disorder-specific treatments.

## Introduction

By the end of 2018, the unprecedented number of 70.8 million people were forcibly displaced worldwide (1). Germany is a frequent destination for asylum seekers from Syria, Iraq, Iran, or African countries (2). Due to stressful or traumatic experiences before or during their flight, asylum seekers frequently suffer from severe psychological distress upon their arrival in a registration and reception center (3, 4). However, post-migration factors, such as an uncertain residency status and unstable social conditions, can also lead to considerable distress. A systematic meta-analytic review by Steel et al. (5) revealed a prevalence rate of 30.6% for posttraumatic stress disorder (PTSD) and 30.8% for depressive disorders among refugees (5). Moreover, in an investigation of the prevalence of mental disorders in asylum seekers in Germany, Bozorgmehr et al. (6) found a range of prevalence rates from 6.7 to 76.7% for PTSD relative to the applied psychometric instruments and examined samples (6).

In a recent study (7), Nikendei et al. investigated the symptom course of mental disorders and the access to psychotherapeutic treatment of asylum seekers in a three month follow-up after transfer from a state registration and reception center to municipal shelters. After three months, symptoms of PTSD and anxiety had not changed significantly. However, while still remaining in a pathological range, depression, panic disorder symptoms, and mental well-being had significantly improved (7). Furthermore, although 66% of the participating asylum seekers had been referred to psychotherapy after their first assessment, none of them had started outpatient psychotherapeutic treatment at follow-up. As most common reasons for not being able to start psychotherapy, participants reported language barriers, long waiting times as well as frequent transfers to other registration and reception centers. However, asylum seekers with mental health problems are able to use other services in the German health care system in addition to psychotherapeutic treatment offers following their transfer to municipal shelters. These services include the use of outpatient psychiatrists, counselling centers for general health advice, and general practitioners (GPs). In this context, previous studies from Germany found that health care services are particularly needed in the early phase after asylum seekers’ arrival in a host country and emphasized the importance of facilitating asylum seekers’ access to the health care system to improve their health status and integration (8–10). Therefore, in order to identify possible predictors of asylum seekers’ health care utilization, the present investigation further analyzed data collected in the recent study by Nikendei et al. (7).

To conceptualize health care utilization in general, Andersen established a behavioral model containing three variables: *predisposition*, *enablement*, and *need* (11, 12). **“**Predisposition**”** refers to sociodemographic factors, such as gender, age, religion, whereas **“**enablement**”** encompasses the availability of services and individual social factors facilitating the use of health care services (13). Finally, **“**need**”** refers to health and disorder related aspects and comprises both objective and subjective indicators for somatic or psychiatric disorders. Previous investigations have frequently focused on the **“**need**”** aspect of this conceptualization and have shown that refugees with mental health problems are more likely to consult GPs than to seek specialized psychological treatment (13–17), especially when compared to health care utilization behavior found in the general population (18). In this context, Laban et al. (13) revealed that poor perceived general health and functional impairment were the most important predictors of health care utilization in asylum seekers. Moreover, McCracken et al. examined predictors of health care utilization in depressive patients and identified the severity of depression, perceived health status, social functioning, and the level of social support as significant factors (19).

In the present study, we aimed to investigate predictors of asylum seekers’ health care utilization, mainly focusing on the *predisposition* and *enablement* components of Andersen’s model. The following groups of variables were investigated as possible predictors: a) sociodemographic variables, like gender, age, number of children, religion, and language proficiency in English or German; b) emotion regulation (ER) and sense of coherence (SOC) and c) the asylum seekers’ psychiatric diagnoses which were assessed at the first measurement point. ER comprises psychological and behavioral processes that allow individuals to control their perception, intensity, duration, and expression of emotion. In the present study, the concept of ER will be used in terms of the two strategies *cognitive reappraisal* and *expressive suppression* (20). Cognitive reappraisal means to actively change the way of perceiving a stressful situation, whereas expressive suppression consists in inhibiting the outward signs of inner feelings (21). Previous studies have suggested that the habitual use of cognitive reappraisal is more likely to predict increased mental well-being and long-term health, while the use of expressive suppression was more likely to be associated with negative indicators of mental health over time (22, 23). SOC is a central aspect of Antonovsky’s concept of salutogenesis and comprises three components, namely, comprehensibility, manageability, and meaningfulness (24, 25). SOC was previously demonstrated to be an essential factor in the development and maintenance of mental health (26). In particular, high degrees in SOC were consistently demonstrated to facilitate an individual’s health-promoting behavior and may consequently enable a psychological burdened individual to actively seek help by health care services (27, 28). Building on previous studies, we therefore hypothesize that (1) sociodemographic factors differentially predict health care utilization of asylum seekers in the early phase of resettlement; (2) ER strategies differentially predict utilization of health care services; (3) high degrees of SOC are a predictor for higher utilization of health care services; and (4) asylum seekers’ psychiatric diagnoses differentially predict subsequent utilization of health care services.

## Material and Methods

The analyzed data of the present study and a part of the applied methodology is based on a recent study by Nikendei et al. (7).

### Study Design

We used a cross-sectional study with two surveys to investigate predictors of the asylum seekers’ utilization of different types of health care services in the early post-migration phase. As early post-migration phase, we defined the period between the asylum seekers’ arrival in the registration and reception center and the first months after transition to municipal shelters. We administered (1) an assessment of sociodemographic and psychometric data as well as psychiatric diagnoses, and (2) a structured interview on the utilization of different health care services which took place three to five months after the first assessment.

### Participants and Eligibility

The study was performed over a period of seven months, from September 2017 until March 2018. It took place in a state registration and reception center for asylum seekers on the premises of the former barracks in Heidelberg-Kirchheim, Germany, named “Patrick Henry Village” (PHV). All asylum seekers who consulted the psychosocial outpatient clinic implemented in PHV (29, 30) and met the inclusion criteria were invited to participate in the present study. The average time between an asylum seeker’s arrival in the registration and reception center PHV and the first consultation at the psychosocial outpatient clinic was found to be 4.8 weeks (SD ± 5.8 weeks) in a previous investigation (30). All participants had applied for asylum in Germany before visiting the psychosocial outpatient clinic. None of the participants had already been granted official refugee status. Inclusion criteria for our study were: consultation of the psychosocial outpatient clinic, fluency in one of 16 most frequently spoken languages in the state registration and reception center PHV (languages see below), and the age 18 years or older. Exclusion criteria were: cognitive disabilities (i.e. mental disability or dissociative stupor), illiteracy, and an age under 18 years.

### Health Care Regulations for Asylum Seekers in Germany

As the German Asylum Seekers’ Benefits Act (“Asylbewerberleistungsgesetz”) primarily aims to ensure the basic treatment and prevention of acute conditions and pain (31), asylum seekers only have limited access to psychotherapeutic care in the early stages of resettlement. However, consultations with outpatient physicians, psychiatrists, or counselling centers for diagnostic evaluations can be approved on a case-by-case-basis by the relevant regional council or social welfare office (32). After being granted official refugee status, asylum seekers are entitled to the same medical treatment services as the rest of the German general population with national health insurance, although interpreters costs are not covered (33).

### Medical Health Care Provision in a State Registration and Reception Center

Besides statutory medical examination procedures for asylum seekers, state registration and reception centers often provide additional basic medical services, for example *via* an onsite physician. However, the PHV provides medical care beyond basic medical services and has established an outpatient clinic for asylum seekers on its premises (29) including subdivisions for general medicine, pediatrics, gynecology, and psychosocial medicine (30). In this outpatient clinic, asylum seekers are provided with health care services by registered physicians from Heidelberg and physicians from Heidelberg University Hospital. In the psychosocial outpatient clinic, medical doctors and psychologists from the Center of Psychosocial Medicine, as part of the University Hospital Heidelberg, offer consultations twice a week (34).

### Recruitment

Asylum seekers waiting for their first consultation in the psychosocial outpatient clinic in PHV (29, 30) were provided with information about the study and were subsequently invited to participate. After obtaining informed consent, participating asylum seekers were asked for their telephone number to enable contacting them for the interview. The asylum seekers were explicitly informed that participation in the study would not affect their asylum procedure or their healthcare in any way. The recruitment, the assessment, and all interviews were conducted by two trained research assistants.

### Ethical Approval

The study was approved by the ethics committee of the University of Heidelberg (S-041/2017) and all participating individuals gave their written informed consent in accordance with the Declaration of Helsinki.

### Cultural Sensitive Translation and Experts’ Discussions

All written materials in this study, including the applied questionnaires, were provided in 16 different languages (English, German, French, Persian, Arabic, Turkish, Kurmanji (Northern Kurdish), Urdu, Hausa, Russian, Serbian, Albanian, Macedonian, Georgian, Mandinka, Tigrinya). Whenever available, we applied validated versions of the questionnaires in the respective language. In absence of a validated version, we translated the questionnaire *via* a standardized translation procedure followed by expert discussion (35): in a first step, the questionnaires were translated independently by two professional translators; then, a third translator created a synthesis of those two versions, which was then discussed by a multidisciplinary expert committee consisting of a medical doctor, a psychologist and an anthropologist. The final version of the respective questionnaire was the result of the experts’ discussion.

### Assessment of Possible Predictors (t1)

Participants were asked for general and cultural background related sociodemographic information (i.e. gender, age, origin, religion/atheism, number of children, and pregnancy). Then, they were invited to fill in questionnaires to assess emotion regulation (ERQ-10) and sense of coherence (SOC-9L). The psychometric questionnaires were presented on a tablet using the QuestionPro ^®^ Survey Software.

#### ERQ-10 (Emotion Regulation Questionnaire)

To assess the participant’s habitual emotion regulation strategies, we applied the ERQ-10 questionnaire (36). This questionnaire comprises 10 items designed to measure a person’s tendency to regulate their emotions either in terms of (1) *expressive suppression* or (2) *cognitive reappraisal*. Each item can be answered on a 7-point Likert-scale ranging from 1 (strongly disagree) to 7 (strongly agree). The ERQ showed acceptable internal consistencies for reappraisal (Cronbach’s alpha = 0.82) and suppression (Cronbach’s alpha = 0.76) (37).

#### SOC-9L (Sense of Coherence Scale, Leipzig Short Scale)

In order to assess an individual’s degree of sense of coherence, we used the SOC-9L (38), which is a short form of the original SOC-29 scale (24). The SOC-9L questionnaire consists of 9 items, with 2 items focusing on comprehensibility, 3 items assessing manageability, and 4 items relating to meaningfulness. The SOC-9L questionnaire displayed good internal consistency (Cronbach’s alpha = 0.87), with a high correlation to the original SOC-29 scale (r=0.94) (38).

#### Psychiatric Diagnoses

Subsequently, the participants’ psychiatric diagnoses given by the medical doctor or psychologist in the context of the consultation in the psychosocial outpatient clinic were taken from the discharge letters that the patients received after their consultation.

### Assessment of Health Care Utilization (t2)

The interviews were conducted *via* telephone and were planned to take place three months after participants’ first consultation in the psychosocial outpatient clinic. If not available the first time, participants were contacted up to ten times before being excluded from the study. As a result, interviews took place between three to five months after assessment of possible predictors (t1). To explore the participants’ utilization of health care services after municipal shelter transfer, we conducted structured interviews. We asked participants about their health care utilization for the time between their municipal shelter transfer and their interview. We asked them whether they had used the following health care services during that time: consultation with outpatient psychiatrists, utilization of counselling centers with the aim to get general medical advice, and consultations with GPs. Non-English or German speaking participants were interviewed with the help of an interpreter.

### Statistical Analysis

We used hierarchical logistic regression analysis using the “MASS” package (39) of the statistic framework R (40) to predict each type of utilization as dichotomous variables on the basis of three blocks of predictors that are added to the models in consecutive steps. In four models we defined (1) the general utilization of services, which is the utilization of at least one type of service, as well as (2) the utilization of psychiatrists, (3) counselling centers, and (4) GPs. The sequential blocks of predictors were (1) sociodemographic variables (gender, age, number of children, religion, language proficiency in English or German), (2) psychological variables (SOC, ER with subscales expressive suppression and cognitive reappraisal); and (3) most frequently assigned clinical diagnosis (PTSD, depression/adaptation disorders, anxiety disorders). This approach makes it possible to determine the proportion of variance (R²) of the criterion variables that are explained by each block. In each model, the partial standardized regression coefficients provide information about the unique strength of the association between the predictor variables and the types of utilization with other predictors held constant. Goodness of fit for the logistic regression models was tested by Hosmer–Lemeshow tests [“hoslem.test” function of the R package ResourceSelection; (41)] where a non-significant *χ²*-test statistic indicates good fit. McFadden´s pseudo *R²* values provided information about the degree of variance explanation by the prediction models. The improvement of the model fit by adding the next block of predictors was tested by likelihood ratio tests [“lrtest” function of the R package lmtest; (42)] where a significant *χ²*-test indicates better fit of the model after adding a block than the intercept only, the first or the second model (incremental validity). For all regression models the variance inflation factor, tested by the “vif” function of the R package car (43), was below the value of 2.73 so that multicollinearity was not an issue.

## Results

### Attrition and Sample Composition

During the study period from September 2017 to March 2018, a total of 355 patients consulted the psychosocial outpatient clinic. Of those, 313 individuals met the inclusion criteria. Of those, overall 263 asylum seekers furthermore gave their written informed consent; this corresponds to a participation rate of 84%. As main reason for declining participation in the investigation, asylum seekers stated to feel too burdened by their symptoms. Baseline assessment was completed by 228 participants. Of those, 66 individuals (28.9%) participated in both the first assessment and the telephone interview. The main reason for this attrition was that the participants were often not available *via* telephone after transfer from the registration and reception center to municipal shelters. Therefore, data on the utilization of health care services could be obtained from 66 participants. The data of one person originating from an “Organization for Economic Co-operation and Development” (OECD) country was excluded from further analysis because they were less representative for the rest of the sample.

Sample characteristics and descriptive statistics of the study variables are shown in **Table 1**. The final sample included 65 individuals (*M* age = 32.2 years, SD = 8.8) and consisted of more males (*n* = 40, 61.5%) than females (n = 25, 38.5%). Approximately 57% of the study participants had no children, 11% one child, 20% two children, and each 6% three and four children. We grouped the countries of origin into three areas: Eastern Europe (Georgia, Albania, Kosovo, and Armenia) with 10.8% of the participants, Asia (Iraq, Syria, Turkey, Kurdistan, Afghanistan, Iran, and Sri Lanka) with 60% of the participants, and Africa (Nigeria, Algeria, Cameron, Tunisia, Gambia, Eritrea, Ghana, and Somalia) with 29.2% of the participants. However, due to the low representativeness, we have not analyzed the continent of origin as predictor in the regression analysis. In terms of religion, 44.6% of the participants reported being Christian, and 44.6% being Muslim. In a third category (10.8%) we summarized *n* =1 Jewish participant, *n* = 3 Atheists, and *n* = 3 people with other religions. A good proficiency in English or German language was reported by *n* = 22 (33.8%) participants.

**Table 1:** Sample characteristics and study variables.

*N* = 65		codes	*n (%)*	*M* (*SD*)	*range*
General utilization		1/0	52 (80)		
**Type of utilization**	Psychiatrist	1/0	22 (33.8)		
	Counselling Centre	1/0	30 (46.2)		
	General practitioner	1/0	32 (49.2)		
**Sex**	Male	0	40 (61.5)		
	Female	1	25 (38.5)		
	Age		32.2 (8.8)	18 - 56	
**No. of children**	0	0	37 (56.9)		
	1	1	7 (10.8)		
	2	2	13 (20.0)	0.9 (1.3)	0 - 4
	3	3	4 (6.2)		
	4	4	4 (6.2)		
**Religion**	Christianity	1	29 (44.6)		
	Islam	2	29 (44.6)		
	Other religion	3	7 (10.8)		
**Continent**	**East Europe** (Georgia, Albania, Kosovo, Armenia)	1	7 (10.8)		
	**Asia** (Iraq, Syria, Turkey, Kurdistan, Afghanistan, Iran, Sri Lanka)	2	39 (60.0)		
	**Africa** (Nigeria, Algeria, Cameron, Tunisia, Gambia, Eritrea, Ghana, Somalia)	3	19 (29.2)		
Language proficiency in English or German		1/0	22 (33.8)		
Sense of coherence				38.4 (6.5)	22 - 54
**ERQ**	Reappraisal			4.2 (1.7)	1 - 7
	Suppression			4.3 (1.5)	1 - 7
**Diagnosis**	PTSD	1/0	32 (49.2)		
	Depression and Adaptation Disorder	1/0	32 (49.2)		
	Anxiety disorders (panic: n = 6; mixed anxiety and depression: n = 6)	1/0	12 (18.5)		

For the analyses of missing data (only six data were missing), we first used Little´s χ2 tests, which provided evidence for the assumption of missing completely at random (MCAR). Missing values could therefore be imputed by the “mice” package (44) of the statistic program R.

### Correlations Between Study Variables and Results of Regression Analysis

Correlations between study variables are shown in **Table 2**. Significant positive correlations were found between number of children on the one side and general utilization of health care services, utilization of GPs and age on the other side; utilization of counselling centers on the one side and SOC and Christian religion on the other side; cognitive reappraisal on the one side and Islam religion, other religion and expressive suppression on the other side; as well as between expressive suppression on the one side and the utilization of GPs, Islam religion, and other religion on the other side. Significant negative correlations were found between other religion on the one side and cognitive reappraisal and expressive suppression on the other side.

**Table 2:** Means, standard deviations, and correlations with confidence intervals.

Variable	*M*	*SD*	1	2	3	4	5	6	7	8	9	10	11	12	13	14	15	16
																		
1. General utilization (1/0)	0.80	0.40																
																		
2. Psychiatrist (1/0)	0.34	0.48	.36**															
																		
3. Counselling center (1/0)	0.46	0.50	.46**	.19														
																		
4. General Practitioner (1/0)	0.49	0.50	.49**	-.05	.08													
																		
5. Gender (0 = male, 1 = female)	0.38	0.49	-.24	-.10	-.16	-.02												
																		
6. Age (18 – 56)	32.16	8.83	.12	.18	-.15	.16	.02											
																		
7. No. of children (0 – 4)	0.94	1.26	.25*	-.02	.05	.29*	.11	.53**										
																		
8. Christian religion (1/0)	0.45	0.50	.14	.08	.29*	-.08	-.01	-.06	-.05									
																		
9. Islam religion (1/0)	0.45	0.50	-.17	-.18	-.15	.11	-.01	.11	.12	-.81**								
																		
10. Other religion (1/0)	0.11	0.31	.05	.17	-.22	-.04	.03	-.07	-.10	-.31*	-.31*							
																		
11. Language (1/0)	0.34	0.48	-.13	.04	.19	-.12	-.16	-.02	-.07	.08	-.05	-.04						
																		
12. Sense of coherence (22 – 54)	38.42	6.48	.23	.08	.31*	.05	-.00	.17	.11	.11	-.01	-.16	.09					
																		
13. ER – reappraisal (1- 7)	4.24	1.67	-.03	.16	.10	-.03	-.21	-.11	-.03	-.07	.25*	-.28*	.10	.10				
																		
14. ER – suppression (1- 7)	4.27	1.51	.01	-.00	.15	.25*	-.00	-.12	-.02	-.09	.30*	-.33**	.06	-.06	.59**			
																		
15. PTSD (1/0)	0.49	0.50	.18	.01	.20	.20	-.08	-.06	.12	-.14	.17	-.04	-.25*	-.02	.10	.21		
																		
16. Depr. & adapt. disorders (1/0)	0.49	0.50	-.05	.01	-.17	.14	.04	.12	-.17	.04	-.14	.15	-.12	-.22	-.12	.01	.02	
																		
17. Anxiety disorders (1/0)	0.25	0.43	-.07	-.18	-.10	.01	.21	.01	.14	-.01	-.01	.03	.04	.08	-.08	-.05	-.13	-.28*
																		

M and SD are used to represent mean and standard deviation, respectively. *indicates p < .05. **indicates p < .01.

The results of the hierarchical regression analysis are shown in **Table 3**. With regard to the model for general utilization, step two with sociodemographic and psychological variables showed best fit with 36% explained variance and the significant predictors: male gender, higher number of children, and SOC. With respect to the model for consultations with psychiatrist, the Hosmer & Lemeshow tests were satisfactory, but the likelihood ratio tests could not prove the better fit of the individual blocks compared to the zero-model with intercept only or the previous model. For this reason, the results cannot be interpreted meaningfully with regard to this criterion variable. For the model of counselling center utilization, the second step with sociodemographic and psychological variables showed the best fit with 25% explained variance. A younger age and higher SOC values proved to be significant predictors. For the model of consultations of GPs, the second step again showed the best fit and explained 19% of the total variance. Strongest influence in this model showed the predictors higher number of children and the ER strategy expressive suppression. In none of the models the last block with the indicators of psychological stress has proved to be significant, showing no statistical fit and little explanation of variance. Thus, specific types of mental stress seem to be less relevant for the use of mental health services.

**Table 3:** Results of the hierarchical logistic regression analyzes (part 1).

*N* = 65	Model 1: General utilization						Model 2: Psychiatrist					
	*βstep 1*		*βstep 2*		*βstep 3*		*βstep 1*		*βstep 2*		*βstep 3*	
	*OR* (95% *CI*)	*β*	*OR* (95% *CI*)	*β*	*OR* (95% *CI*)	*β*	*OR* (95% *CI*)	*β*	*OR* (95% *CI*)	*β*	*OR* (95% *CI*)	*β*
*Gender*	0.19 (0.04,0.78)	–.83*	0.07 (0.01,0.45)	–1.33**	0.08 (0.01,0.61)	–1.22*	0.63 (0.2,2.02)	-.22	0.85 (0.25,2.94)	-0.08	1.02 (0.28,3.68)	.01
*Age*	0.99 (0.9,1.07)	–.13	0.99 (0.89,1.10)	–.06	0.99 (0.89,1.12)	–.01	1.07 (1,1.16)	.62†	1.09 (1.01,1.18)	0.76*	1.1 (1.01,1.21)	.88*
*No. of children*	2.42 (0.97,6)	1.11†	3.48 (1.07,11.31)	1.56*	3.08 (1,9.5)	1.42*	0.8 (0.47,1.37)	-.27	0.76 (0.43,1.32)	-0.35	0.69 (0.37,1.3)	-.46
*Religion* (RC: Christianity)												
Islam	0.33 (0.07,1.52)	–.55	0.19 (0.03,1.32)	.84†	0.16 (0.02,1.21)	–.91†	0.42 (0.12,1.41)	-.44	0.3 (0.08,1.15)	-0.60†	0.27 (0.07,1.1)	.65†
Other religion	1.12 (0.08,15.03)	.04	1.12 (0.08,15.03)	.17	1.59 (0.1,24.17)	.15	1.55 (0.3,7.9)	.14	2.22 (0.35,14.04)	0.26	3.09 (0.41,23.12)	.37
*Language*	0.36 (0.08,1.56)	–.49	1.67 (0.12,24.18)	–.54	0.36 (0.06,2.24)	–.48	1.04 (0.33,3.29)	.02	0.95 (0.29,3.16)	0.02	1.1 (0.28,3.61)	.00
*Sense of coherence*			1.19 (1.02,1.39)	1.14*	1.18 (1.01,1.38)	1.07*			1.01 (0.92,1.12)	0.07	1.02 (0.92,1.14)	.13
*Emotion regulation – ERQ*												
reappraisal			0.68 (0.35,1.32)	–.65	0.67 (0.34,1.33)	–.66			1.55 (0.96,2.51)	0.73†	1.53 (0.93,2.5)	.70†
suppression			2.25 (0.99,5.14)	1.23†	2.07 (0.89,4.83)	1.10†			0.94 (0.57,1.53)	-0.09	0.95 (0.56,1.63)	-.07
*Diagnosis*												
PTSD					1.9 (0.32,11.42)	.32					1.25 (0.35,4.51)	.11
Depr. & Adaptation					0.73 (0.11,4.58)	–.16					0.44 (0.1,1.87)	-.42
Anxiety disorders					0.67 (0.08,5.67)	–.17					0.28 (0.05,1.54)	-.55
												
*Model summary*												
HL test: *χ²(df* = 8)	5.62, *p* =.69		4.99, *p* =.76		6.42, *p* =.60		8.54, *p* =.38		3.13, *p* =.93		3.12, *p* =.93	
McFadden´s pseudo *R²*	.22		.36		.37		.08		.14		.17	
* ΔR²*	.22		.14		.1		.08		.6		.3	
LR test: *χ²(df)*	14.40 (6)*		9.17 (3)*		0.68 (3)		6.82 (6)		4.57 (3)		3.16 (3)	
VIF range	1.08 – 1.33		1.18 – 2.71		1.23 – 2.73		1.04 – 1.43		1.05 – 1.91		1.13 – 1.89	

OR, odds ratio with CI = 95% confidence interval; Standardized regression coefficients and model tests are significant at ^†^p < .1, *p < .05, **p < .01, ***p < .001; Language: German or English. RC, reference category for contrast; LR, Likelihood ratio test for comparing with null-model/previous model; HL = Hosmer and Lemeshow test (non-significant = good fit).

**Table 3:** Results of the hierarchical logistic regression analyzes (part 2).

*N* = 65	Model 3: Counselling center						Model 4: General Practitioner					
	*βstep 1*		*βstep 2*		*βstep 3*		*βstep 1*		*βstep 2*		*βstep 3*	
	*OR* (95% *CI*)	*β*	*OR* (95% *CI*)	*β*	*OR* (95% *CI*)	*β*	*OR* (95% *CI*)	*β*	*OR* (95% *CI*)	*β*	*OR* (95% *CI*)	*β*
*Gender*	0.45 (0.14,1.42)	–.39	0.32 (0.09,1.2)	–.56†	0.42 (0.1,1.7)	–.42	0.71 (0.24,2.14)	–.16	0.47 (0.14,1.63)	–.37	0.42 (0.11,1.59)	–0.42
*Age*	0.92 (0.84,1.01)	–.71†	0.9 (0.8,1)	–.98*	0.9 (0.8,1.02)	–.90	1.00 (0.93,1.07)	.01	1.00 (0.93,1.08)	.06	0.99 (0.91,1.07)	–0.10
*No. of children*	1.63 (0.91,2.94)	.62	1.71 (0.88,3.33)	.68	1.61 (0.77,3.33)	.59†	1.68 (0.98,2.87)	.65	1.81 (1,3.27)	.74*	2.1 (1.07,4.12)	0.93*
*Religion* (RC: Christianity)												
Islam	0.34 (0.11,1.11)	–.53†	0.3 (0.08,1.13)	–.60†	0.23 (0.05,0.98)	–.74*	1.28 (0.42,3.86)	.12	1.09 (0.32,3.74)	.04	1.13 (0.31,4.05)	0.06
Other religion	0.18 (0.03,1.17)	–.56†	0.2 (0.02,1.89)	–.54	0.27 (0.03,2.37)	–.43	0.7 (0.13,3.7)	–.12	1.27 (0.2,7.89)	.08	0.95 (0.14,6.57)	–0.017
*Language*	2.33 (0.73,7.42)	.40	2.42 (0.67,8.79)	.42	3.27 (0.8,13.4)	.56	0.6 (0.2,1.85)	–.24†	0.52 (0.15,1.82)	–.31	0.59 (0.16,2.23)	–0.25
*Sense of coherence*			1.16 (1.04,1.29)	.95**	1.16 (1.03,1.3)	.96*			1.04 (0.95,1.14)	.25	1.06 (0.96,1.17)	0.35
*Emotion regulation – ERQ*												
reappraisal			0.77 (0.49,1.24)	–.43	0.78 (0.48,1.26)	–.41			0.63 (0.4,1)	–.77†	0.64 (0.4,1.03)	–0.75†
suppression			1.55 (0.89,2.7)	.66	1.48 (0.83,2.64)	.59			2.11 (1.24,3.59)	1.14**	2.05 (1.17,3.57)	1.08*
*Diagnosis*												
PTSD					3.08 (0.76,12.44)	.57					1.53 (0.45,5.25)	0.22
Depr. & Adaptation					0.7 (0.17,2.86)	–.18					3.18 (0.81,12.51)	0.58†
Anxiety disorders					0.43 (0.09,2.01)	–.37					1.47 (0.31,6.92)	0.17
												
*Model summary*												
HL test: *χ²(df* = 8)	5.98, *p* =.65		10.29, *p* =.24		7.17, *p* =.52		9.55, *p* =.23		10.68, *p* =.22		3.99, *p* =.86	
McFadden´s pseudo *R²*	.13		.25		.29		.08		.19		.22	
* ΔR²*	.13		.12		.4		.08		.11		.03	
LR test: *χ²(df)*	12.53 (6)†		9.65 (3)*		4.02 (3)		7.49 (6)		9.27 (3)*		3.25 (3)	
VIF range	1.06 – 1.97		1.23 – 2.39		1.26 – 2.65		1.00 – 1.37		1.07 – 2.00		1.12 – 2.08	

OR, odds ratio with CI = 95% confidence interval; Standardized regression coefficients and model tests are significant at ^†^p < .1, *p < .05, **p < .01, ***p < .001; Language: German or English. RC, reference category for contrast; LR, Likelihood ratio test for comparing with null-model/previous model; HL, Hosmer and Lemeshow test (non-significant = good fit).

## Discussion

In the present study, predictors of health care utilization among asylum seekers in the early phase of resettlement were investigated. In regard to the general utilization of health care services, male gender, a higher number of children, and high degrees in SOC were indicated to be predictors. For the asylum seekers’ utilization of counselling centers, a younger age and high degrees of SOC were revealed to be predictive. Consultations with GPs were predicted by a higher number of children and the use of expressive suppression as a frequent strategy of emotion regulation. However, mental health diagnoses were not shown to be predictive for subsequent health care utilization. According to Andersen’s health care utilization model, encompassing the three variables *predisposition*, *enablement*, and *need*, mental health diagnoses are subsumed under the *need* variable (11, 12). Therefore, the *need* aspect seems not to predict asylum seekers’ health care utilization in the early stages of resettlement. This surprising observation may be interpreted in different ways. Firstly, predisposing and enabling factors of health care utilization may have such high psychosocial relevance for asylum seekers in the early phase of the asylum process, which is characterized by considerable social and legal uncertainties, that mental health diagnoses become less influential for subsequent utilization of health care. Secondly, our finding may also be interpreted using the concept of health literacy, which describes a general set of skills that an individual needs to function in the health care environment (45–47). In a 2014 study, Wangdahl et al. found inadequate or limited levels of health literacy among a majority of refugees in Sweden (48). Difficulties due to limited health literacy may be associated with a lack of knowledge about mental disorders and possible psychotherapeutic or psychiatric treatment options in general (49–51). In addition, culture-specific differences in perceiving of and coping with mental health problems were shown to play an important role for asylum seekers’ health care (52) and could explain why psychiatric diagnoses were not found to be predictive for health care utilization in the present study. In the following, we would like to highlight the individual predictors identified by the statistical analysis in regard to the asylum seekers’ utilization of health care services. The individual predictors partly accounted for the utilization of different health care services at the same time. Therefore, we have structured the discussion in a predictor-centered manner.

### Sociodemographic Factors

In the analysis, we found that a higher number of children is a predictor for asylum seekers’ general utilization of health care services and for consultations with GPs. This result is in accordance with previous studies emphazising that acculturation processes take place within the context of families, with children quickly seeking contact to the new culture, while parents are often characterized as “lagging behind” their children in adopting cultural competencies and language proficiency (53, 54). In this way, children may facilitate access to the new culture for their parents and, thus, pave the way for utilization of health care. Moreover, our findings may also be explained by the parents’ increased sense of responsibility towards their children in terms of maintaining their health, especially in the uncertain and foreign environment of the host country.

In the present study, a younger age was revealed to be a predictor for asylum seekers’ utilization of counselling centers. In this context, younger asylum seekers might rather seek general health advice first in counselling centers, before consulting with psychiatrists or GPs. In particular, they may be more reluctant towards consultations with psychiatrists and might have more prejudices with regard to psychiatric medication. In a 2019 study, Biddle et al. however found that in particular young and male asylum seekers showed a higher health status on the one hand and lower health care service utilization on the other hand in comparison to female and older asylum seekers (55). To further explore the effects of asylum seekers’ age on mental health care utilization in the early stages of resettlement, more research is needed.

Male gender was found to be a predictor for general health care utilization in the early phase of resettlement. At first sight, this finding seems to be contrary to the results of previous studies, which had consistently revealed female gender to be associated with higher utilization of medical services (56–58). In a 2011 study, Weiss et al. analyzed data from the United Nations High Commissioner of Refugees (UNHCR) Health Information System (HIS), which contains data on refugee settlements in Africa, Asia, and the Middle East (59). This investigation indicated that female refugees in refugee settlements had a statistically significant higher utilization of health care services than male refugees (59). In the present study, however, we examined health care utilization of asylum seekers, who were already living in municipal shelters in their host country. Against this background, a possible explanation for our findings could be that female asylum seekers may be more reluctant to use outpatient services in a country that is still foreign for them, whereas male asylum seekers may be more active in the early post-migration phase. However, more research is needed to further understand the different aspects of gender differences in health care utilization among asylum seekers.

### Sense of Coherence and Emotion Regulation

SOC was identified as a predictor for the asylum seekers’ general utilization of health care services as well as for the utilization of counselling centers. This seems to be an intuitive finding, since the SOC is defined by the three components comprehensibility, manageability, and meaningfulness (24, 26). The aspect of manageability can be assumed to be of particular relevance for the utilization of health care services because it points to an individual’s perception of being able to actively address and solve their problems effectively. In the context of the present study, this could mean that asylum seekers with a high degree of SOC are not resigned to their fate of being psychologically burdened or traumatized, but actively try to solve problems by seeking health care services.

The habitual use of expressive suppression as a strategy of emotion regulation was demonstrated to be a predictor for the consultation with GPs. This finding is in accordance to a theoretical psychosomatic framework which identifies suppression of emotional processes to be a key step in the somatization of symptoms (60). In this context, the expression of aversive emotions is suppressed to a degree that they eventually only find their manifestation in the form of somatic symptoms. This process may lead to the consultation of a GP instead of a psychiatrist or counselling center (61–63).

Nevertheless, it is surprising that ER strategies were only able to explain a limited percentage of the variance in health care utilization in asylum seekers. However, when interpreting these results it is important to bear in mind the background of our study: we examined health care utilization during the early phase of resettlement which is characterized by considerable social instabilities and uncertainties regarding asylum procedure and residence status. In this early postmigration phase, ER strategies seem to play only a limited role in the utilization of health care. A possible interpretation of this finding is, that the socially and legally uncertain context may overburden effects of ER strategies promoting health care utilization. However, it can be assumed that these psychological variables may display stronger effects on health care utilization on longer time scales.

### Consequences for Asylum Seekers’ Care

With regard to Andersen’s model of health care utilization, the present study mainly identified factors of predisposition and enablement for health care service utilization. It was demonstrated that specific sociodemographic factors, such as gender, age, and number of children on the one hand, and the differential use of ER strategies and different degrees of SOC, on the other hand, can be predictive for the utilization of health care services. Asylum seekers with a corresponding “risk profile” for lower utilization of health care should therefore be identified early on in order to facilitate their access to mental health care. Comprehensive psychoeducation should be implemented at an early stage in order to inform asylum seekers about mental health disorders, the different services within the health care system of the host country, and to address reservations and fears towards the utilization of such offers. Furthermore, low-threshold treatment offers for asylum seekers should be increasingly established in registration and reception centers, in order to identify and treat mental disorders at an early stage of resettlement and to prevent further chronification of the conditions. Especially in the early phase of resettlement, more culturally sensitive health care services need to be implemented to address asylum seekers’ different cultural peculiarities sufficiently. Given the cultural relativity of psychopathology and established treatment approaches, GPs will continue to be important health care system gatekeepers even after the asylum seekers’ transfer to municipal shelters. GPs therefore hold the important tasks of recognizing mental health disorders, informing and motivating asylum seekers about and for psychotherapeutic or psychiatric treatment as well as finally referring them to indicated specialist treatment.

## Limitations

Several limitations of the presented study should be addressed: first of all, our investigation is limited by the relatively small number of participants, resulting in poorer model fit and reducing generalizability of the results. The considerable loss to the telephone interview may be explained by the unstable living conditions in the early phase after migration. The participants’ telephone reachability and access was often restricted in this context. Hence, our mental health care utilization predictor analysis may be influenced by an attrition bias. Moreover, carry-over effects cannot be ruled out because the pre-post assessments were performed by the same research assistants. Although this study explicitly focused on the early phase after migration, the short period to the second assessment after 3–5 month could also be a limitation of the predictor analysis. However, a longer period of time to the telephone interview would have most likely resulted in an even higher attrition as even fewer participants would have been available due to the unstable nature of their living arrangements. Furthermore, the participating asylum seekers’ heterogeneity may limit our findings as individual, culture specific aspects of mental health care utilization could not be addressed accordingly. Apart from that, a selection bias cannot be ruled out with regard to the following aspects: 1.) we exclusively recruited psychologically burdened asylum seekers who attended the psychosocial outpatient clinic; these asylum seekers may furthermore be assumed to have a basic openness towards mental health issues; 2.) since some asylum seekers declined participation, because they felt too burdened by their symptoms, we cannot preclude the possibility that included participants might be psychologically less burdened; 3.) we merely included literate asylum seekers, which furthermore had to be fluent in one of the 16 selected languages in the state registration and reception center. This selection bias may reduce generalizability of our results. Finally, the present study only focused on a selection of possible predictors and their influence on a selected number of health care offers. However, other predictors may also be associated with the utilization of health care services. Future studies should take into account further health care offers for asylum seekers on the one hand and the quality of received health care services for asylum seekers on the other hand.

## Conclusions

Individual sociodemographic factors, such as gender, age, and number of children, as well as the emotion regulation strategy of expressive suppression and sense of coherence were shown to be predictive for the utilization of health care services among asylum seekers. These effects may be specific for the early phase of resettlement. Accordingly, low-threshold, culture-sensitive treatment options for asylum seekers should be increasingly established at an early stage. GPs are important health care gatekeepers for asylum seekers and should act as a central hub for further referrals to disorder-specific treatments.

## Data Availability Statement

The datasets used and analyzed during the present study are available from the corresponding author upon reasonable request.

## Ethics Statement

The studies involving human participants were reviewed and approved by Ethics committee of the University of Heidelberg (S-041/2017). The patients/participants provided their written informed consent to participate in this study.

## Author Contributions

DK and CN conceived the study. DK, CN, VZ, KB, and EN participated in the design of the study. VZ and DK carried out the study. CN, KB, and H-CF supervised the project. EN and DK carried out the quantitative analysis. DK and EN finally drafted the manuscript. All authors read and approved the final manuscript.

## Funding

This study was supported by the Ministry of Science, Research and Arts, Baden-Württemberg, Germany (project identification No.D100011720; AZ42-04 HV.MED (16)27/1) and by Ruprecht-Karls-Universität Heidelberg.

## Conflict of Interest

The authors declare that the research was conducted in the absence of any commercial or financial relationships that could be construed as a potential conflict of interest.

The reviewer JD declared a shared affiliation, with no collaboration, with one of the authors, CN, to the handling editor.
